# Reducing cardiac implantable electronic device–induced artefacts in cardiac magnetic resonance imaging

**DOI:** 10.1007/s00330-022-09059-w

**Published:** 2022-08-27

**Authors:** Aino-Maija Vuorinen, Lauri Lehmonen, Jarkko Karvonen, Miia Holmström, Sari Kivistö, Touko Kaasalainen

**Affiliations:** 1 Radiology, HUS Diagnostic Center, University of Helsinki and Helsinki University Hospital, P.O. Box 340, HUS, 00029 Helsinki, Finland; 2 Heart and Lung Center, University of Helsinki and Helsinki University Hospital, P.O. Box 340, HUS, 00029 Helsinki, Finland

**Keywords:** Magnetic resonance imaging, Pacemaker, Artificial, Defibrillators, Implantable, Artefacts, Electrodes, Implanted

## Abstract

**Objectives:**

Cardiac implantable electronic device (CIED)–induced metal artefacts possibly significantly diminish the diagnostic value of magnetic resonance imaging (MRI), particularly cardiac MR (CMR). Right-sided generator implantation, wideband late-gadolinium enhancement (LGE) technique and raising the ipsilateral arm to the generator during CMR scanning may reduce the CIED-induced image artefacts. We assessed the impact of generator location and the arm-raised imaging position on the CIED-induced artefacts in CMR.

**Methods:**

We included all clinically indicated CMRs performed on patients with normal cardiac anatomy and a permanent CIED with endocardial pacing leads between November 2011 and October 2019 in our institution (*n* = 171). We analysed cine and LGE sequences using the American Heart Association 17-segment model for the presence of artefacts.

**Results:**

Right-sided generator implantation and arm-raised imaging associated with a significantly increased number of artefact-free segments. In patients with a right-sided pacemaker, the median percentage of artefact-free segments in short-axis balanced steady-state free precession LGE was 93.8% (IQR 9.4%, *n* = 53) compared with 78.1% (IQR 20.3%, *n* = 58) for left-sided pacemaker (*p* < 0.001). In patients with a left-sided implantable cardioverter-defibrillator, the median percentage of artefact-free segments reached 87.5% (IQR 6.3%, *n* = 9) using arm-raised imaging, which fell to 62.5% (IQR 34.4%, *n* = 9) using arm-down imaging in spoiled gradient echo short-axis cine (*p* = 0.02).

**Conclusions:**

Arm-raised imaging represents a straightforward method to reduce CMR artefacts in patients with left-sided generators and can be used alongside other image quality improvement methods. Right-sided generator implantation could be considered in CIED patients requiring subsequent CMR imaging to ensure sufficient image quality.

**Key Points:**

*• Cardiac implantable electronic device (CIED)–induced metal artefacts may significantly diminish the diagnostic value of an MRI, particularly in cardiac MRIs.*

*• Raising the ipsilateral arm relative to the CIED generator is a cost-free, straightforward method to significantly reduce CIED-induced artefacts on cardiac MRIs in patients with a left-sided generator.*

*• Right-sided generator implantation reduces artefacts compared with left-sided implantation and could be considered in CIED patients requiring subsequent cardiac MRIs to ensure adequate image quality in the future.*

**Supplementary Information:**

The online version contains supplementary material available at 10.1007/s00330-022-09059-w.

## Introduction

Cardiac magnetic resonance (CMR) imaging is often the preferred imaging method for the advanced evaluation of the heart, allowing for the non-invasive assessment of cardiac function, structure, myocardial scars and hemodynamics [1]. The clinical impact of CMR imaging is significant also in CIED patients [2–4]. The current European Society of Cardiology Guidelines on cardiac pacing approve magnetic resonance imaging (MRI) in patients with a cardiac implantable electronic device (CIED), such as pacemakers (PMs), implantable cardioverter-defibrillators (ICDs) and cardiac resynchronization therapy (CRT) devices [5]. However, CIEDs induce local magnetic field inhomogeneities due to the metallic materials used in the CIED generators and pacing leads resulting in susceptibility-based artefacts on MRI [6, 7]. These artefacts appear as signal loss areas and geometric distortions in the images. Moreover, when using balanced steady-state free precession (bSSFP) sequences for cine or late-gadolinium enhancement (LGE) imaging, any ferromagnetic material in or near the field of view increases the presence of banding artefacts in the images. These CIED-induced image artefacts may significantly degrade the image quality (IQ) and diagnostic value of MRIs, especially in the areas close to the generator, such as the heart [8–11].

Several methods for reducing CIED-related artefacts have been proposed in recent studies, such as right-sided generator implantation and wideband LGE imaging [8, 12, 13]. More generally, metal artefacts can be reduced by using wideband bSSFP pulse sequences, a frequency-scout method prior to the bSSFP cine and LGE imaging, and spoiled gradient echo (SPGR) sequences instead of bSSFP [14, 15].

**Fig. 1:**
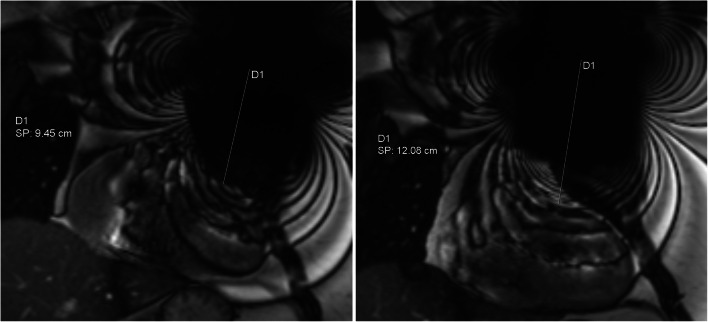
CMR coronal bSSFP localiser with the arm-down (left) and arm-raised (right) scanning position. The shortest distance from the middle of the signal-void area artefact induced by the CIED generator to the border of the heart was measured in cm. Arm-raised scanning increases the distance between the heart and the generator. D1 indicates the distance measured and SP shows the measurement result in cm

In addition, elevating the ipsilateral arm relative to the CIED generator during MRI has been described as a part of CIED patient MRI scanning protocols [16]. Raising the arm on the side of the generator increases the distance between the heart and the generator (Fig. 1). This method has been used to decrease CIED-induced artefacts overlaying the myocardium [6, 16, 17]. However, the effect of arm-raised imaging on CMR IQ remains unknown.

In this retrospective study, we aimed to report on CMR IQ and safety in patients with CIED and to assess the impact of arm-raised imaging and generator implantation site on the CIED-induced artefacts.

## Materials and methods

This retrospective study of MRIs from patients with CIED was approved by the Helsinki University Hospital Medical Imaging Center review board. No informed consent was required. The data cannot be made available to other researchers for the purposes of reproducing the results, given the restrictions imposed by the research permissions. Individual-level data cannot be shared openly.

### Study population

We evaluated all CMRs performed on adult patients with CIED at Helsinki University Hospital between November 2011 and October 2019 (*n* = 260). All CMRs were clinically indicated, except for one CMR, which was scanned as a part of a clinical study. Patients with congenital heart disease (*n* = 60), epicardial pacing leads (*n* = 2), subcutaneous ICDs (*n* = 2), temporary permanent pacemaker systems (*n* = 24) and for whom CMR image data were absent (*n* = 1) were excluded. All CMRs in patients with a permanent CIED and endocardial pacing leads and available CMR image data were included in this study (*n* = 171).

### Data collected from the electronic medical records

The following information was collected from patient electronic medical records (EMRs): patient date of birth, sex, CIED generator model, date of the CIED implantation, site of the CIED generator implantation, date of the CMR scan and information on the pacing device interrogation prior to and after the CMR scan. EMRs were searched specifically for any CIED-related safety hazards or adverse outcomes during or after the CMR scan, such as generator failure, power-on reset, clinically relevant changes in the pacing threshold or sensing that required system revision or programming changes, unexpected battery depletion and pacing inhibition and patient-reported events, such as discomfort, pain, a warm sensation at the location of the device and palpitations.

### CMR protocol

All CMR examinations were performed using a 1.5T system (Siemens MAGNETOM Avanto, which in summer 2013 was upgraded to a Siemens MAGNETOM Avanto^fit^ (both from Siemens Healthcare). The CMR scans were performed following our institutional MRI in a CIED patient safety protocol presented in detail earlier [18, 19]. The CMR examinations were performed with variable protocols. Considering the CMR imaging indication, the appropriate scanning protocol was selected case by case. Typically, a CMR scan consisted of localiser imaging, cines, a T2-weighted turbo spin echo, modified Look-Locker inversion recovery T1 mapping, T2 mapping, rest perfusion and late-gadolinium-enhanced (LGE) imaging sequences. For cine imaging, bSSFP (TrueFISP) or SPGR (FLASH) sequences were used. According to our institutional protocols, bSSFP sequences are primarily used for cine and LGE image acquisition. However, SPGR sequences are used when large, CIED-induced artefacts are present over the myocardium already in the localiser images or in the first scanned bSSFP cine images. The used contrast agent was gadoteric acid—gadoterate meglumine (Dotarem® 279.3 mg/ml, Guerbet). The typical dose of contrast agent administration was 0.4 ml/kg.

### CMR IQ assessment

**Fig. 2:**
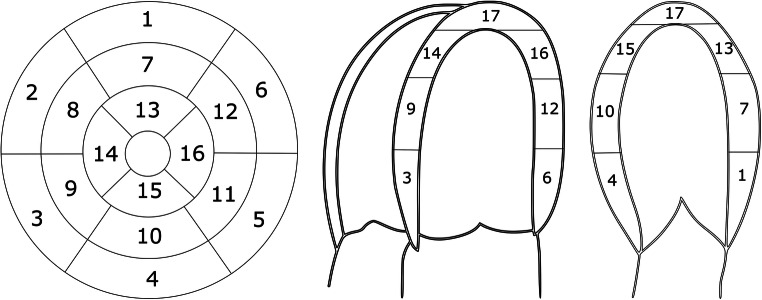
Short-axis, four-chamber (4ch) and two-chamber (2ch) views of the left ventricle myocardium and segment distribution based on the American Heart Association (AHA) 17-segment model

Selected sequences of the included CMR examinations were evaluated for the presence of artefacts; cine and LGE, both the conventional inversion recovery and phase-sensitive inversion recovery (PSIR) images acquired using bSSFP or SPGR techniques, were evaluated along three planes: short axis (SAX), four-chamber (4ch) and two-chamber (2ch) (Fig. 2). We used the American Heart Association (AHA) 17-segment model to detect regional differences in artefacts [20]. In the cine, conventional LGE and PSIR images, the left ventricular (LV) segments were evaluated for the presence of CIED-related artefacts (yes/no) through consensus among two radiologists (one experienced radiologist, specialised in CMR with over 10 years of experience, and one radiology resident also familiar with CMR examinations). The area (cm^2^) of the susceptibility-related artefact induced by the right ventricular (RV) pacing lead tip was measured in SAX sequences. In the same manner, the LV coronary sinus lead-induced artefact area (cm^2^) was measured in CRT patients. In addition, the largest diameter (cm) of the signal-void artefact caused by the CIED generator was measured from the bSSFP localiser images. The distance from the generator to the heart was measured using the coronal localiser images of the CMR (the shortest distance from the middle of the signal-void area artefact induced by the CIED generator to the border of the heart, cm). The longest distance from the middle of the signal-void artefact area to any banding artefact induced by the CIED generator was also measured using the coronal bSSFP localiser images. Variability in selected measured artefact variables (generator-induced signal-void artefact diameter, distance from the generator to the heart, distance of the banding artefact and RV lead tip–induced artefact area in SAX bSSFP and SPGR sequences) between two expert observers was evaluated in a small sample (*n* = 25 CMR examinations).

### Statistical analysis

Numerical results are given as mean ± standard deviation (SD) or median (interquartile range [IQR]), as appropriate. Variable comparisons between several groups were calculated using the Kruskal-Wallis test applying the post hoc Dunn test with Bonferroni’s adjustment. The Mann-Whitney *U*-test was used for pairwise comparisons. *p* values < 0.05 were considered statistically significant. The interobserver variability was assessed using an intraclass correlation coefficient (ICC) with a 95% confidence interval (CI). Absolute agreement using the two-way mixed model was calculated. ICC < 0.50 was considered poor. Data were analysed using SPSS (IBM Corp. Released 2013. IBM SPSS Statistics for Windows, Version 25.0).

## Results

### Baseline characteristics

In total, 171 CMR examinations from 167 patients with CIED (85 females, 50.9%) were included in this study. The patients’ mean age at the time of MRI was 58.8 ± 13.2 years (range 22–84 years). The implantation of the CIED generator was performed on average 2.6 ± 2.7 years (range 0–12.5 years) prior to the CMR imaging. All generators were implanted in the year 2005 or later. The time of implantation was missing in three cases. Four patients underwent two CMR scans. The majority (78.9%) of the CMR examinations were performed on patients with a PM, while 12.9% of patients had an ICD and 8.2% had a CRT device, respectively (Table 1). Most generators of the CIEDs (60.2%, *n* = 103) were classified as MR unsafe models according to the MR Task group of the American Society for Testing and Materials (ASTM) International [21, 22]. The data on CIED MR compatibility was missing in two cases (1.2%). Seven patients underwent a CMR with an abandoned endocardial pacing lead, and additionally, one patient had an abandoned PM system in addition to the functioning CIED system.

**Table 1:**
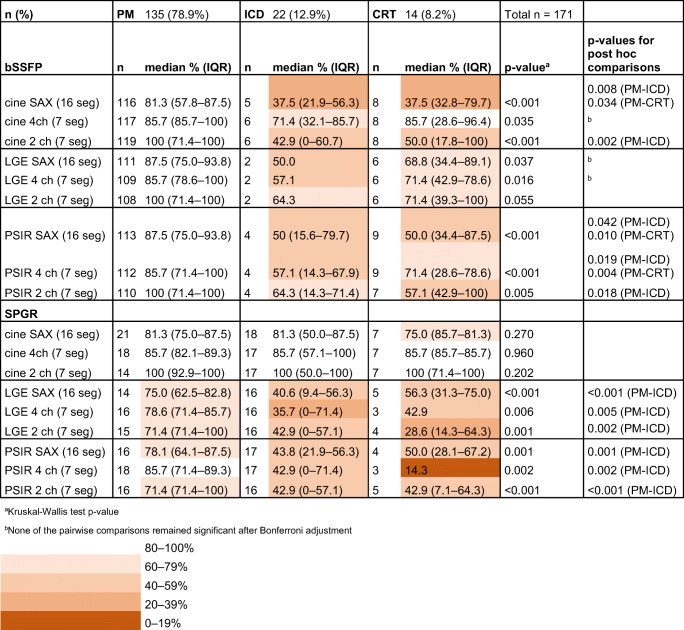
Artefact-free segments per CIED model and sequence type

*bSSFP* balanced steady-state free precession, *CIED* cardiac implantable electronic device, *CRT* cardiac resynchronization therapy device, *ICD* implantable cardioverter-defibrillator, *IQR* interquartile range, *LGE* late-gadolinium enhancement, *PM* pacemaker, *PSIR* phase-sensitive inversion recovery, *SAX* short axis, *SPGR* spoiled gradient echo, *2ch* two-chamber, *4ch* four-chamber

### CMR imaging safety in patients with CIED

Adverse events were detected in three CMR examinations (3/171, 1.8%). These consisted of an unintended change to the CIED pacing mode, an ICD beeper malfunction and a reversible elevation of the pacing threshold. CMRs in patients with abandoned leads and abandoned PM systems were performed without detectable adverse events.

The CMR was prematurely interrupted in two patients. In a patient with a CRT-D, the CMR was interrupted after a few SPGR cine sequences due to the major CIED-induced artefacts and insufficient IQ. One patient with a PM had a large amount of pleural fluid leading to an ECG gating problem, and the CMR was aborted.

In one PM-dependent patient, the PM (generator model Medtronic Kappa KSR 401) was programmed to asynchronous pacing mode (DOO) before the MRI examination. However, during the MRI, the PM reached the elective replacement indicator (ERI) mode due to temporarily programmed high output voltage, which led to an inhibited pacing mode (VVI). After the MRI, the PM was reprogrammed, and its function reverted to normal. In one patient, the audible alarm failure of an ICD was noted following the MRI (ICD generator model: Boston Scientific Autogen EL VR). The beeper malfunction did not affect the function of the ICD. A reversible elevation of the pacing threshold was detected in another patient with an ICD (Medtronic Evera MRI DVMC3D4 generator and 6947M Sprint Quattro Secure 62 cm lead). In this patient, the pacing threshold 1.5 years before the MRI was 0.75V/0.4ms, while immediately following the MRI, the pacing threshold was elevated to 2.25V/0.4 ms or 1.5V/0.8 ms, which required reprogramming of the device (the pulse width was increased to 0.8 ms). The pacing threshold values immediately before the MRI were missing in the EMR. Ten months after the MRI, the pacing threshold was decreased to 1.625V/0.4 ms, and 2.5 years later, the pacing threshold was 1.25V/0.4 ms.

### CIED-induced artefacts on CMR

**Fig. 3:**
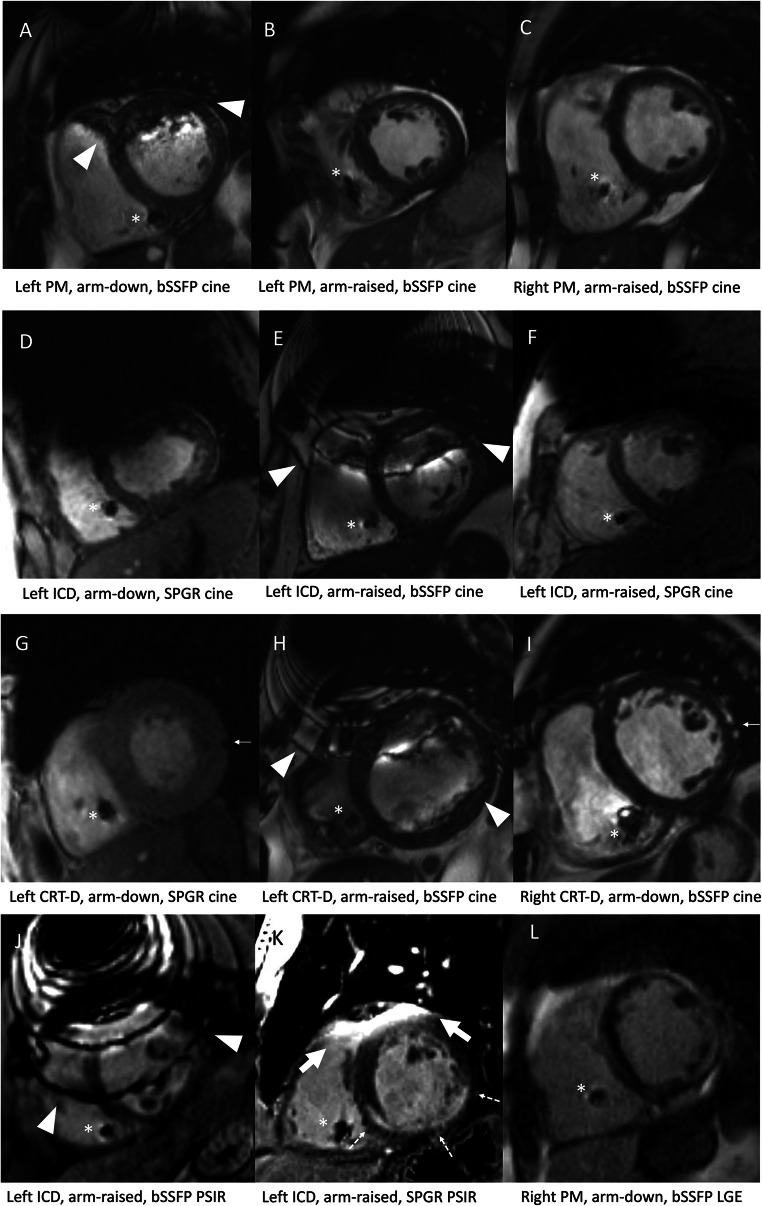
Typical appearances of CIED-related artefacts on CMR images. All images are in short-axis plane. The RV lead-induced artefacts are indicated with an asterisk (**A**–**L**). Banding artefacts seen in bSSFP sequences are presented with arrowheads (**A**, **E**, **H**, **J**). The thick arrow shows hyperintensity artefact, typically seen in SPGR sequences (**K**). The thin arrow indicates the LV coronary sinus lead-induced artefact (**G**, **I**). Generator-induced banding artefact interfering with the myocardium is seen in bSSFP sequences, typically in patients with left-sided generator (**A**, **E**, **H**, **J**). In SPGR sequences, a large signal-void area induced by the generator is a typical artefact (**D**, **F**). Arm-raised scanning position can shift the generator-induced artefacts away from the myocardium (**B**, **F**). However, severe artefacts may be present despite the arm-raised scanning position (**E**, **H**, **J**). In patients with left-sided CRT device, LV lead-induced artefact can be obscured by generator-induced artefacts (**H**). Hyperintensity artefact in LGE imaging could be misinterpreted as positive LGE (**K**). In image **K**, the LV anterior wall is affected by hyperintensity artefact, but the subendocardial LGE finding in the LV inferior wall related to ischemic scar is clearly visible. In patients with right-sided generator, the image quality is usually diagnostic (**C**, **I**, **L**). Image **L** shows patchy intramyocardial and subepicardial LGE in the LV wall extending to the RV wall, related to cardiac sarcoidosis. bSSFP = balanced steady-state free precession; CIED = cardiac implantable electronic device; CMR = cardiac magnetic resonance imaging; CRT-D = cardiac resynchronization therapy; CRT-D = cardiac resynchronization therapy defibrillator; ICD = implantable cardioverter-defibrillator; LGE = late-gadolinium enhancement; LV = left ventricular; PM = pacemaker; PSIR = pPhase-sensitive inversion recovery; RV = right ventricular; SPGR = spoiled gradient echo

**Fig. 4:**
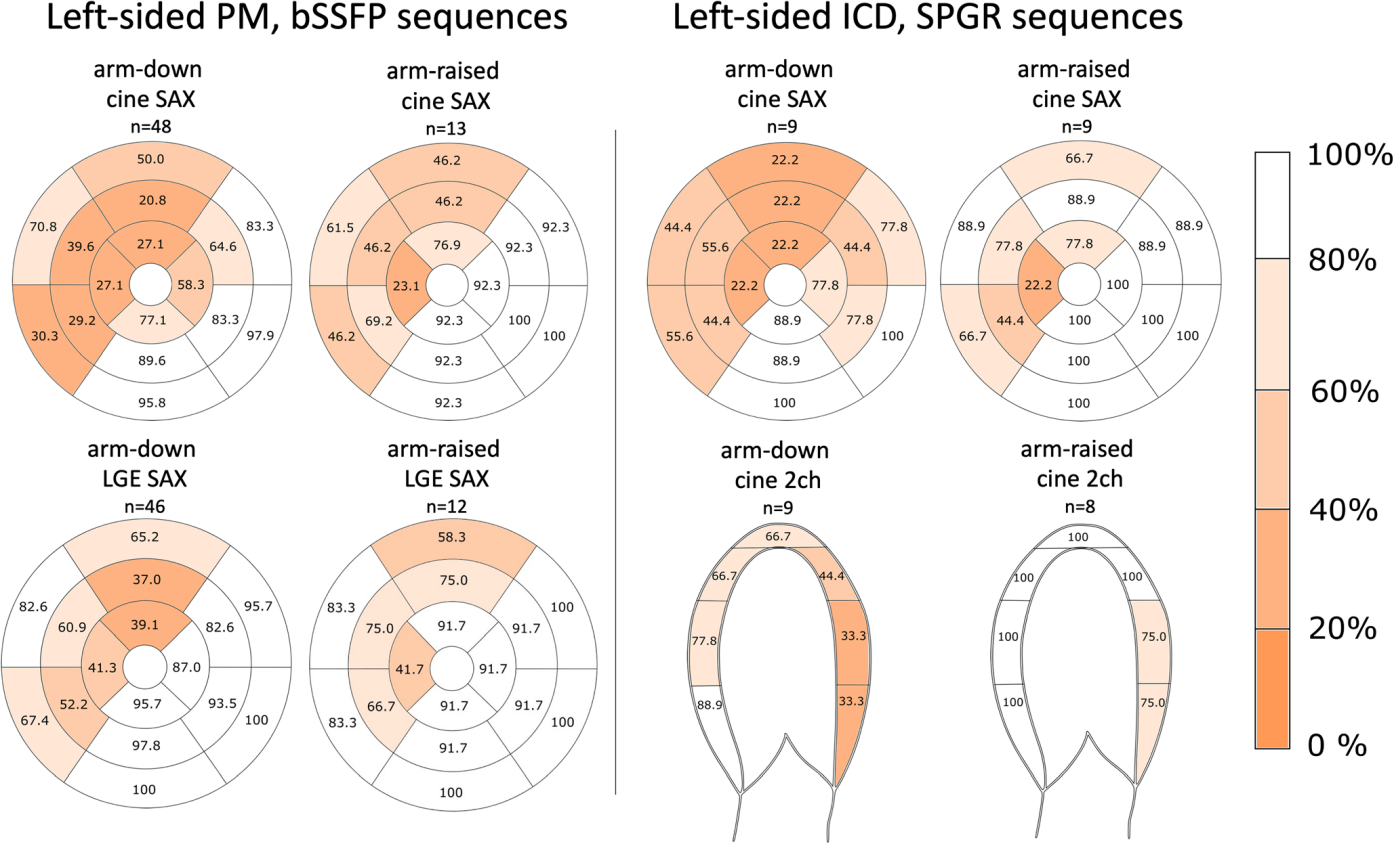
The prevalence and distribution of artefact-free segments as percentage with arm-down and arm-raised scanning position in patients with left-sided PM (on the left) and ICD (on the right). In patients with PM, the IQ of the bSSFP cine and LGE SAX sequences are illustrated; in patients with ICD, the SPGR cine in SAX and 2 ch planes are illustrated respectively. CIED-induced artefact distribution shows that the anterior wall and septal segments are most affected by the artefacts. bSSFP = balanced steady-state free precession; CIED = cardiac implantable electronic device; CMR = cardiac magnetic resonance imaging; ICD = implantable cardioverter-defibrillator; LGE = late-gadolinium enhancement; PM = pacemaker; SAX = short axis; SPGR = spoiled gradient echo; 2ch = two-chamber

The analysis of CIED-induced CMR artefacts per segment revealed that patients with ICD or CRTs had more artefacts than patients with PM in almost all evaluated sequences (Table 1). Description of artefact-free segments in CRT-D and CRT-P groups separately is provided in Supplementary Table 1. Typical appearances of the CIED-induced artefacts on CMR are presented in Fig. 3. The diameter of the susceptibility artefact (i.e. the signal loss area) caused by the CIED generator was substantially larger with ICDs than with PMs (Table 2). However, we detected no significant difference in the artefact area induced by the RV lead tip between the CMRs with PMs and ICDs (Table 3). The LV coronary sinus lead-induced artefact was measured in CRT patients (*n* = 14). LV lead-induced artefact could not be measured in two CRT patients: no image data in SAX planes were available in one patient. In another patient, the LV lead-induced artefact was not distinguishable from the generator-induced artefacts. The medians of measured LV lead-induced artefact areas in bSSFP sequences were as follows: cine SAX 0.34 (0.31–0.51) cm^2^ (*n* = 7), LGE SAX 0.38 cm^2^ (*n* = 3), PSIR SAX 0.37 (0.28–0.47) cm^2^ (*n* = 5). Similarly, the medians in SPGR sequences were as follows: cine SAX 0.38 (0.31–0.67) cm^2^ (*n* = 7), LGE SAX 0.32 (0.27–0.65) cm^2^ (*n* = 5), PSIR SAX 0.37 (0.31–0.51) cm^2^ (*n* = 5); the distribution of CIED-induced artefacts in patients with a left-sided PM or ICD in selected sequences is presented in Fig. 4. The anterior wall and septal segments of the LV were the most affected by artefacts. The interobserver variability in the analysed variables ranged from excellent to poor. The best correlations were obtained in the generator artefact diameter, ICC 0.84 (CI 0.84–0.85), and the generator distance from the heart, ICC 0.87 (CI 0.87–0.88), while the RV lead artefact area in bSSFP PSIR, SPGR PSIR and LGE images had a poor agreement (ICC < 0.50) between the observers (Suppl. Table 2).

**Table 2:** Artefacts induced by the generator per CIED model

*n* (%)	PM	135 (78.9%)	ICD	22 (12.9%)	CRT-D	9 (5.3%)	CRT-P	5 (2.9%)	Total *n* = 171	
	*n*	Median (IQR)	*n*	Median (IQR)	*n*	Median (IQR)	*n*	Median (IQR)	*p* value^a^	*p* values of post hoc comparisons
Susceptibility signal-void artefact diameter measured from coronal localiser (bSSFP), cm	126	8.9 (7.9–9.8)	19	13.6 (12.6–14.5)	7	14.1 (13.0–14.8)	5	9.5 (9.0–9.7)	< 0.001	< 0.001 (PM–ICD) < 0.001 (PM–CRT-D) 0.045 (CRT-P–ICD)
Banding artefact distance measured from coronal localiser (bSSFP), cm	126	10.4 (8.9–12.3)	19	20.6 (18.2–21.7)	7	18.8 (18.2–24.6)	5	11.8 (10.3–13.3)	< 0.001	< 0.001 (PM–ICD) < 0.001 (PM–CRT-D)
Generator distance from the heart, measured from coronal localiser (bSSFP), cm	126	10.3 (8.6–12.0)	19	10.0 (8.5–11.9)	7	10.5 (7.9–11.3)	5	7.3 (6.5–10.7)	0.341	

^a^Kruskal-Wallis test *p* value

*bSSFP* balanced steady-state free precession

*CIED* cardiac implantable electronic device

*CRT-D* cardiac resynchronization therapy defibrillator

*CRT-P* cardiac resynchronization therapy pacemaker

*ICD* implantable cardioverter-defibrillator

*LGE* late-gadolinium enhancement

*IQR* interquartile range

*PM* pacemaker

**Table 3:** Right ventricular pacing lead-induced artefact area on CMR per CIED model and sequence type

*n* (%)	PM	135 (78.9%)	ICD	22 (12.9%)	CRT-D	9 (5.3%)	CRT-P	5 (2.9%)	Total *n* = 171
	*n*	Median (IQR)	*n*	Median (IQR)	*n*	Median (IQR)	*n*	Median (IQR)	*p* value
bSSFP									
Cine SAX RV lead artefact area (cm^2^)	115	1.6 (1.2–2.1)	5	1.9 (1.1–2.3)	4	2.3 (2.1–3.5)	4	1.6 (1.1–1.8)	0.118
LGE SAX RV lead artefact area (cm^2^)	110	1.6 (1.3–2.1)	2	1.2	5	1.9 (1.9–2.2)	1	1.1	0.160
PSIR SAX RV lead artefact area (cm^2^)	113	1.6 (1.2–2.1)	4	1.2 (0.9–1.7)	6	1.9 (1.5–2.4)	3	1.1	0.283
SPGR									
Cine SAX RV lead artefact area (cm^2^)	21	1.8 (1.3–2.4)	18	1.4 (1.1–1.8)	4	1.4 (1.3–2.3)	3	2.0	0.083
LGE SAX RV lead artefact area (cm^2^)	14	2.1 (1.3–2.7)	16	1.8 (1.4–2.1)	3	2.3	2	2.5	0.399
PSIR SAX RV lead artefact area (cm^2^)	16	1.8 (1.3–2.5)	16	1.8 (1.2–2.3)	2	1.6	2	1.3	0.759

*bSSFP* balanced steady-state free precession

*CIED* cardiac implantable electronic device

*CMR* cardiac magnetic resonance imaging

*CRT-D* cardiac resynchronization therapy defibrillator

*CRT-P* cardiac resynchronization therapy pacemaker

*ICD* implantable cardioverter-defibrillator

*LGE* late-gadolinium enhancement

*PM* pacemaker

*PSIR* phase-sensitive inversion recovery

*RV* right ventricular

*SAX* short axis

*SPGR* spoiled gradient echo

### Generator location and artefacts

The measured generator distance from the heart was significantly greater in patients with right-sided generators (11.2 ± 2.3 cm, CI 10.5–11.8 cm, *n* = 56) compared with patients with left-sided generators (9.6 ± 2.9 cm, CI 9.0–10.1 cm, *n* = 101); *p* < 0.001. Right-sided generator implantation significantly reduced the presence of artefacts in CMRs with a PM in all analysed bSSFP images (Table 4). However, the number of artefact-free segments in CMRs also remained higher in patients with left-sided PMs than in patients with left-sided ICDs in all evaluated SPGR LGE and PSIR planes (*p* ≤ 0.002). All ICDs and CRT-Ps were implanted on the left side.

**Table 4:**
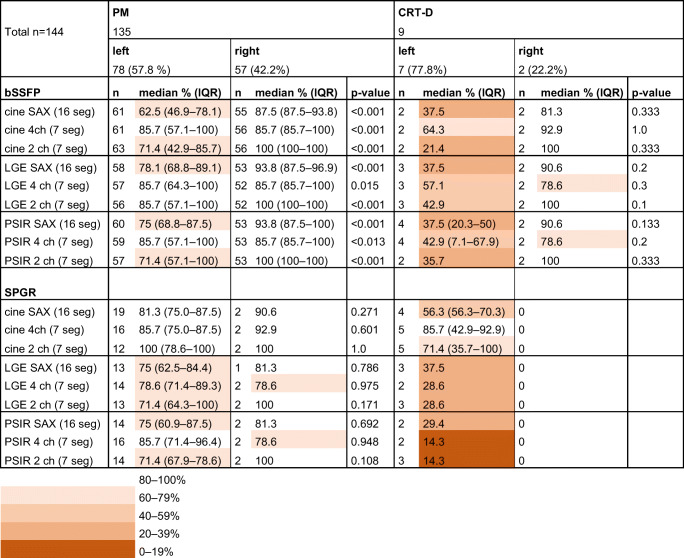
CIED-induced artefacts on CMR by CIED generator implantation site (left/right)

*bSSFP* balanced steady-state free precession, *CIED* cardiac implantable electronic device, *CMR* cardiac magnetic resonance, *CRT-D* cardiac resynchronization therapy defibrillator, *LGE* late-gadolinium enhancement, *PM* pacemaker, *PSIR* phase-sensitive inversion recovery, *RV* right ventricular, *SAX* short axis, *SPGR* spoiled gradient echo, *2ch* two-chamber, *4ch* four-chamber

### Arm-raised imaging and artefacts

In patients with a left-sided generator, the measured distance between the generator and the heart was significantly greater in patients with the arm-raised CMR scanning position (mean 11.0 ± 2.1 cm, CI 10.2–11.8, *n* = 28) than in patients with arm-down scanning position (mean 9.0 ± 3.0 cm, CI 8.3–9.7, *n* = 73); *p* < 0.001. In patients with a left-sided PM, the arm-raised CMR scanning position significantly increased the number of artefact-free segments on several bSSFP image stacks, thereby improving the IQ compared with arm-down scanning (Table 5). Furthermore, in patients with ICD, the arm-raised CMR scanning position improved the IQ significantly in the SPGR SAX cine and 2ch cine sequences (Table 5, Fig. 4).

**Table 5:**
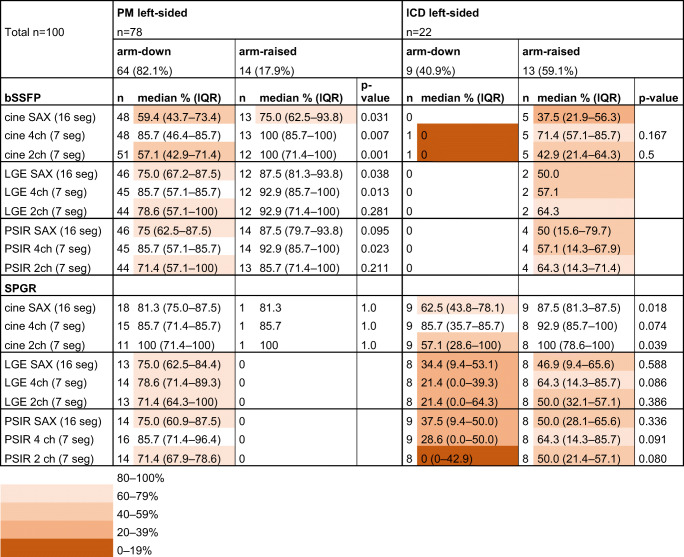
Artefact-free segments on CMR with left-sided CIED generator by scanning position (arm-down/arm-raised)

*bSSFP* balanced steady-state free precession, *CIED* cardiac implantable electronic device, *CMR* cardiac magnetic resonance imaging, *LGE* late-gadolinium enhancement, *PM* pacemaker, *PSIR* phase-sensitive inversion recovery, *SAX* short axis, *SPGR* spoiled gradient echo, *2ch* two-chamber, *4ch* four-chamber

## Discussion

We assessed the CMR IQ of patients with CIED. Our key finding was that the arm-raised imaging reduced CIED-induced artefacts, thus improving the IQ in patients with left-sided PM and ICD. Arm-raised CMR scanning is a simple and cost-free way of diminishing CIED-induced artefacts interfering with the myocardium and can be used alongside other artefact-reducing methods, such as wideband LGE image acquisition. We also noted that right-sided generator implantation improved the number of artefact-free LV segments on CMR in patients with PM. This observation was intuitive since the CIED generator remained outside the scanning field of view and the distance between the generator and the myocardium was greater than that achieved with left-sided generator implantation. The impact of CIED generator location on CMR IQ should be considered when CIED implantation is required for a patient with conditions requiring subsequent CMRs.

### CMR safety in patients with CIED

Numerous large-scale studies have demonstrated the safety of thoracic MRIs in CIED patients [19, 23–26]. The low adverse event rate related to CMR scanning the CIED patients (1.8%) in our study is in line with the published literature. One patient in our patient cohort experienced a potentially catastrophic adverse event involving an unintended change to the CIED pacing mode during the MRI from the asynchronous pacing mode to the inhibited pacing mode in a PM-dependent patient with a Medtronic Kappa PM KSR 401generator. One study reported that older Medtronic Kappa PM models appear more likely to experience a power-on reset during MRI suggesting special attention may be needed with CIED patients undergoing MRI with these PM models [27]. Fortunately, the PM reverted to normal function after reprogramming, and inappropriate pacing inhibition leading to asystole due to electromagnetic interference did not occur in this reported case, which has already been described in our previous publication [19]. In addition, an ICD beeper malfunction was noticed after the MRI in one patient. In the current Boston Scientific ICD models, the beeper is located outside of the generator’s protective cover. Thus, the malfunctioning of the beeper represents an expected possible adverse event related to an MRI in these specific devices [28]. Importantly, the beeper malfunction does not affect the functioning of the ICD and, thus, does not carry a risk to patient safety. The third event was a transient elevation of the pacing threshold following a CMR detected in a patient with an ICD. This likely resulted from the lead heating up during MRI, causing oedema in the pacing lead tip area tissues. The CIED reprogramming was required. During follow-up, the pacing threshold reverted to the pre-MRI level. There was no need for CIED revision in this case. Similar transient and persistent elevations in the pacing threshold related to MRIs in patients with CIED have been reported in previous publications [2, 26, 29, 30].

The generators in our study population were considered modern (implanted in the year 2005 or later), but most of the generators (60.2%) were classified as MR unsafe models. The safety hazards of an MRI are considered more significant for patients with conventional CIEDs implanted before 2001, especially ICDs [31–33]. Notably, a recent large-scale study provides evidence that supports MR conditional labelling of all endocardial PM and defibrillator leads [33]. In clinical practice, the risks of MRI in CIED patients appear to be associated with factors related to the generator and not the leads [33]. Seven patients with abandoned endocardial pacing leads and one patient with an abandoned PM system were CMR scanned without adverse events. This finding is in keeping with the previous studies reporting low adverse event rates related to MRIs in patients with abandoned pacing leads [2, 19, 26, 34, 35].

### CMR in patients with CIED: clinical significance and IQ

Based on previous studies, the clinical impact of CMR imaging on patients with CIED is high. CMR imaging data altered the diagnosis in 35% of the cases and confirmed the diagnosis in 54% of the cases in a study on the clinical impact and MRI safety in patients with MR unsafe CIEDs and abandoned leads [2]. Another study on the utility of CMR imaging in patients with ICD and electrical instability or worsening heart failure symptoms revealed that the CMR examination data changed the diagnosis in 40% of the cases, and management-changing information was gained in 21% of the cases [3]. Despite the potential benefits, CIED patients are underrepresented in MRI examinations [36, 37].

The CIED-induced artefacts may compromise the CMR IQ. Especially, ICDs and CRTs containing more ferromagnetic material than PMs result in more severe artefacts, often threatening the diagnostic value of CMR. An ICD may result in artefacts on CMR in > 50% of the LV segments [9, 10]. The anterior wall of the LV is the most affected area by the ICD-induced artefacts [38]. Our observations support these findings. In contrast, the RV pacing lead-induced artefacts have been shown to be minor and only mildly affecting the image interpretation [8, 39, 40]. Our study showed non-significant differences in the RV lead-induced artefact areas between subgroups of patients with different CIED types supporting that generator-related artefacts are the main factor for IQ differences. However, RV lead-induced artefact area measurements were non-reproducible in bSSFP PSIR, SPGR LGE and SPGR PSIR sequences indicating these results were non-reliable. To our best knowledge, previously, no data of LV coronary sinus lead-induced artefacts on CMR have been published. The measured LV lead-induced artefact areas seem minor in our study with a limited number of CRT patients. In general, the diameter of LV leads is smaller than that of RV leads [41]. Therefore, it seems reasonable that LV lead-induced artefacts do not significantly limit the interpretability of CMR images. Several factors affect the magnitude of the susceptibility artefacts, such as the magnetic susceptibility of the metal, the strength of the main magnetic field, the orientation of the ferromagnetic implant with respect to the main magnetic field, readout direction and the bandwidth [42, 43]. In addition, under a higher magnetic field strength, a greater field inhomogeneity effect of the implants results in larger artefacts. Thus, in general, 1.5T scanners provide fewer metal artefacts than higher field strength MRI scanners [43].

Methods to reduce CIED-related artefacts have been developed. Recent literature has well described that wideband bSSFP sequences, especially in terms of the wideband LGE acquisition, improve IQ in CIED patients [14, 25, 44–46]. Furthermore, using SPGR sequences instead of bSSFP for CMR scanning is beneficial in CIED patients but leads to a lower contrast between the myocardium and blood pool [8, 12, 47]. Moreover, a hyperintensity artefact on SPGR images may lead to difficulties in distinguishing between a myocardial scar and an artefact [7, 48, 49]. Right-sided generator implantation increases the distance between the heart and the generator, thereby reducing the CIED-induced artefacts compared with left-sided generator implantation [4]. Importantly, left-sided generator implantation is preferred with ICDs and CRT-Ds to ensure a low defibrillation threshold [50, 51]. Movement of the CIED generator away from the heart by raising the ipsilateral arm to the generator during CMR scanning has been previously described as an artefact reduction method [6, 16]. However, there is a lack of published literature concerning the actual effect of this method on CMR artefacts.

Our study revealed that arm-raised scanning genuinely improves CMR IQ in patients with a left-sided PM or ICD. This is an effective and straightforward method to decrease artefacts on CMR in CIED patients and may provide a significant clinical benefit. It should be noted, however, that CMR imaging requires being still for a reasonably long time, and arm-raised scanning may lead to patient discomfort and may not be suitable for all patients. In particular, patients with poor physical condition and patients with limited upper extremity function, e.g. due to joint conditions, may not be suitable candidates for arm-raised scanning position. We also noted that patients with a right-sided PM had significantly fewer artefacts on bSSFP sequences than patients with a left-sided PM, which is in line with previously published findings [4, 39]. Considering our findings and the existing literature, device-dependent modifications to the CMR scanning strategy likely improve IQ [47]. Taking the CIED type (PM/ICD/CRT) and the generator site (left/right) into account, the appropriate CMR scanning position (arm-down/arm-raised) and the CMR sequence type (i.e. bSSFP, SPGR and wideband bSSFP technique) could be modified.

## Limitations

We must note several limitations to this study. CRT and ICD subgroups are limited in size in some of the tests. In the comparison of arm-raised and arm-down CMR scanning positions on SPGR IQ of ICD patients, the subgroups included less than 10 patients. Similarly, the CRT-D subgroups included 5 or fewer patients in the comparison of the IQ between left-sided and right-sided generators. The small sample sizes limit the large-scale generalizability of the results. Thus, studies with larger subgroups are needed to confirm the effectiveness of arm-raised scanning position as an artefact reduction method in CMR scanning of the CIED patients. However, to our knowledge, there are no previous publications on the effect of arm-raised imaging on CMR IQ in CIED patients, and we find our results promising. According to our safety protocol, only clinically significant changes in CIED parameter values are entered in the EMR. The data on the exact CIED parameter values were not retrospectively available. Thus, a statistical analysis of the parameter values before and after the CMR was not possible. In addition, we did not assess the CMR IQ of the right ventricle in CIED patients in our study. However, the findings of the right ventricle are of great interest, especially in selected cases, such as in the suspicion of arrhythmogenic right ventricular cardiomyopathy. Wideband LGE acquisition was not used in our institution. Furthermore, we did not assess the IQ in the T1 and T2 mapping sequences. The measurement of the artefact area and distances were mostly performed by one radiologist. However, interobserver variability was assessed in a small sample, which showed that measurements are potentially subjective and include inaccuracies. All CMRs were performed with a 1.5T scanner, and the results cannot be extrapolated to 3.0T scans.

## Conclusions

CIED-related artefacts may significantly reduce the diagnostic value of CMR. Arm-raised imaging is a straightforward, costless method for reducing CIED-induced CMR artefacts in patients with left-sided generators, which can be used alongside other IQ improvement methods, such as wideband LGE. When clinically indicated, right-sided generator implantation should be considered in CIED patients known to require subsequent CMRs in order to ensure adequate IQ in the future.

## Supplementary information

ESM 1 (DOCX 19.1 kb)

## Abbreviations

2ch: Two-chamber
4ch: Four-chamber
AHA: American Heart Association
bSSFP: Balanced steady-state free precession
CIED: Cardiac implantable electronic device
CMR: Cardiac magnetic resonance
CRT: Cardiac resynchronization therapy
CRT-D: Cardiac resynchronization therapy defibrillator
CRT-P: Cardiac resynchronization therapy pacemaker
EMR: Electronic medical record
ERI: Elective replacement indicator
ICD: Implantable cardioverter-defibrillator
IQ: Image quality
LGE: Late-gadolinium enhancement
LV: Left ventricular
PM: Pacemaker
PSIR: Phase-sensitive inversion recovery
RV: Right ventricular
SAX: Short axis
SPGR: Spoiled gradient echo

## Declarations

### Guarantor

The scientific guarantor of this publication is Touko Kaasalainen.

### Statistics and Biometry

No complex statistical methods were necessary for this paper.

### Study subjects or cohorts overlap

Some study subjects or cohorts have been previously reported in *Vuorinen AM, Pakarinen S, Jaakkola I, Holmstrom M, Kivisto S, Kaasalainen T (2019) Clinical experience of magnetic resonance imaging in patients with cardiac pacing devices: unrestricted patient population. Acta Radiol 60:1414-1421.* One hundred twelve cardiac MRIs in were included in this previous publication and are also included in this study. However, our previous article concentrated on the general safety of MRI in patients with CIED and on the MRI safety protocol. This publication concentrates specifically on the CMR image quality and artefacts in CIED patients and adds valuable information in this field.

### Methodology

• retrospective

• observational

• performed at one institution
