# Supplementary material for: Reducing cardiac implantable electronic device–induced artefacts in cardiac magnetic resonance imaging
Source: Eur Radiol. 2022 Aug 27;33(2):1229–42. doi: 10.1007/s00330-022-09059-w (PMC9889467; doi:10.1007/s00330-022-09059-w)
Supplement: Supplementary file 1 — (DOCX 19.1 kb) [file 330_2022_9059_MOESM1_ESM.docx]

| **Supplementary Table 1. Artifact-free segments per CRT and sequence type.** | | | | |
| --- | --- | --- | --- | --- |
| **n (%) Total n = 14** | **CRT-D** | 9 (64.3%) | **CRT-P** | 5 (35.7%) |
| **bSSFP** | **n** | **median % (IQR)** | **n** | **median % (IQR)** |
| cine SAX (16 seg) | 4 | 56.3 (37.5–84.4) | 4 | 34.4 (26.5–70.3) |
| cine 4ch (7 seg) | 4 | 92.9 (42.9–100) | 4 | 57.1 (7.1–85.7) |
| cine 2 ch (7 seg) | 4 | 64.3 (17.8–100) | 4 | 50.0 (21.4–89.3) |
| LGE SAX (16 seg) | 5 | 50.0 (32.3–90.6) | 1 | 87.5 |
| LGE 4 ch (7 seg) | 5 | 71.4 (42.9–78.6) | 1 | 87.5 |
| LGE 2 ch (7 seg) | 5 | 42.9 (35.7–100) | 1 | 100 |
| PSIR SAX (16 seg) | 6 | 50.0 (23.4–89.1) | 3 | 50.0 |
| PSIR 4 ch (7 seg) | 6 | 64.3 (21.4–75.0) | 3 | 71.4 |
| PSIR 2 ch (7 seg) | 4 | 71.4 (32.1–100) | 3 | 57.1 |
| **SPGR** |  |  |  |  |
| cine SAX (16 seg) | 4 | 56.3 (56.3–70.3) | 3 | 81.3 |
| cine 4ch (7 seg) | 5 | 85.7 (42.9–92.9) | 2 | 85.7 (85.7–85.7) |
| cine 2 ch (7 seg) | 5 | 71.4 (35.7–100) | 2 | 100 (100–100) |
| LGE SAX (16 seg) | 3 | 37.5 | 2 | 75.0 |
| LGE 4 ch (7 seg) | 2 | 28.6 | 1 | 85.7 |
| LGE 2 ch (7 seg) | 3 | 14.3 | 1 | 71.4 |
| PSIR SAX (16 seg) | 2 | 31.3 | 2 | 65.6 |
| PSIR 4 ch (7 seg) | 3 | 14.3 | 1 | 85.7 |
| PSIR 2 ch (7 seg) | 2 | 14.3 | 2 | 64.3 |
|  | 80–100% | |  |  |
|  | 60–79% | |  |  |
|  | 40–59% | |  |  |
|  | 20–39% | |  |  |
|  | 0–19% | |  |  |

bSSFP = Balanced steady-state free precession

CRT-D = Cardiac resynchronization therapy defibrillator

CRT-P = Cardiac resynchronization therapy pacemaker

IQR = Interquartile range

LGE = Late gadolinium enhancement

PSIR = Phase-sensitive inversion recovery

SAX = Short axis

SPGR = Spoiled gradient echo

2ch = Two-chamber

4ch = Four-chamber

| **Supplementary Table 2. Interobserver variability in selected measured artefact variables.** | | |
| --- | --- | --- |
| **Variable** | **n^a^** | **ICC (95% CI)** |
| Generator-induced artefact diameter, coronal localiser (cm) | 23 | 0.84 (0.84–0.85) |
| Banding artefact distance, coronal localiser (cm) | 23 | 0.62 (0.58–0.61) |
| Generator distance from heart, coronal localiser (cm) | 23 | 0.87 (0.87–0.88) |
| RV lead-induced artefact area, bSSFP cine (cm^2^) | 16 | 0.59 (0.59–0.61) |
| RV lead-induced artefact area, bSSFP LGE (cm^2^) | 11 | 0.55 (0.50–0.54) |
| RV lead-induced artefact area, bSSFP PSIR (cm^2^) | 13 | 0.46 (0.44–0.48) |
| RV lead-induced artefact area, SPGR cine (cm^2^) | 9 | 0.67 (0.66–0.69) |
| RV lead-induced artefact area, SPGR LGE (cm^2^) | 10 | 0.33 (0.31–0.35) |
| RV lead-induced artefact area, SPGR PSIR (cm^2^) | 8 | 0.04 (0.00–0.08) |

^a^In total, the artefacts of 25 CMR examinations were measured by two radiologists and intraclass correlation coefficient was calculated. Because the CMR scans consisted of variable sequences, all measurements could not be performed in every CMR scan. N represents the number of the artefacts measured by two radiologists in the specific sequence.

bSSFP = Balanced steady-state free precession

CI = 95% confidence interval

ICC = Intraclass correlation coefficient

LGE = Late gadolinium enhancement

PSIR = Phase-sensitive inversion recovery

RV = Right ventricular

SPGR = Spoiled gradient echo
